# Wood−Derived Polymers from Olefin−Functionalized Lignin and Ethyl Cellulose via Thiol–Ene Click Chemistry

**DOI:** 10.3390/polym15081923

**Published:** 2023-04-18

**Authors:** Rongrong An, Chengguo Liu, Jun Wang, Puyou Jia

**Affiliations:** 1 School of Geographic and Biologic Information, Nanjing University of Posts and Telecommunications, Nanjing 210023, China; 2 Institute of Chemical Industry of Forest Products, Chinese Academy of Forestry, 16 Suojin North Road, Nanjing 210042, China

**Keywords:** lignin, ethyl cellulose, cross−linked polymer, thiol–ene click chemistry

## Abstract

Lignin and cellulose derivatives have vast potential to be applied in polymer materials. The preparation of cellulose and lignin derivatives through esterification modification is an important method to endow cellulose and lignin with good reactivity, processability and functionality. In this study, ethyl cellulose and lignin are modified via esterification to prepare olefin−functionalized ethyl cellulose and lignin, which are further used to prepare cellulose and lignin cross−linker polymers via thiol–ene click chemistry. The results show that the olefin group concentration in olefin−functionalized ethyl cellulose and lignin reached 2.8096 mmol/g and 3.7000 mmol/g. The tensile stress at break of the cellulose cross−linked polymers reached 23.59 MPa. The gradual enhancement in mechanical properties is positively correlated with the olefin group concentration. The existence of ester groups in the cross−linked polymers and degradation products makes them more thermally stable. In addition, the microstructure and pyrolysis gas composition are also investigated in this paper. This research is of vast significance to the chemical modification and practical application of lignin and cellulose.

## 1. Introduction

With the increasing depletion of fossil resources and the increasing environmental problems of “white pollution” and “microplastics” caused by the extensive use of non−degradable petroleum−based polymers, the use of bio−based raw materials, instead of petroleum−based compound raw materials, to prepare polymer materials has attracted widespread attention [1]. Cellulose has become an ideal raw material for bio−based polymer materials due to its rich sources, low price, excellent biodegradability, easy modification and many other advantages. Cellulose and its derivatives have been widely used in the fiber, paper, film, plastics, coatings and other industrial fields [2,3,4]. However, due to the existence of a large number of hydrogen bonds within and between the molecules of natural cellulose, the complexity of cellulose aggregation structure and high crystallinity, cellulose is insoluble in water and general organic solvents, in addition to not being able of being melted or processed as traditional plastics, which severely limits the application of cellulose materials. The chemical modification of cellulose includes oxidation, esterification, etherification and other grafting methods [5,6,7]. At present, the esterification derivatives of cellulose include cellulose acetate, cellulose propionate, cellulose butyrate and various cellulose−mixed esters, which are widely used in plastics, coatings, separation membranes, cigarette filters and other daily necessities [8,9,10].

Lignin is a rich natural resource. Compared with other biomass products, lignin has a relatively complex structure and contains a variety of functional groups [11]. It has broad research prospects for the development of appropriate methods for separating and extracting lignin, and then prepare functional composites. At present, lignin has been applied to the preparation of high−value materials, such as porous carbon materials, adsorption materials, capacitor electrode materials, graphene materials, surfactants and hydrogels, and has broad application prospects in the energy, medical, construction, agriculture and other fields [12,13,14,15,16]. The structural unit of lignin is similar to that of phenol. Preparing lignin−based phenolic resin by partially replacing phenol with lignin is the most feasible method [16,17,18,19,20]. Lignin and its derivatives can be used to synthesize bipolar plate materials for phenolic resin fuel cells, phenolic resin catalysts, phenolic resin foams, phenolic resin adhesives and other phenolic resin materials [21,22,23]. Since the 20th century, researchers have been exploring the preparation of lignin−based phenolic resin by replacing phenol with structurally modified lignin derivatives. After the 1990s, the preparation technology of lignin−based phenolic resin developed rapidly because many kinds of structurally modified lignin derivatives with flame retardancy were produced. However, due to the shortcomings of high pollution, high energy consumption, and a complex preparation process, lignin−based phenolic resin cannot be produced on a large scale.

As a simple C–S bonding reaction, “thiol–ene” reaction was discovered over 100 years ago [24]. This reaction has very attractive advantages: First, the C–S bonding reaction can be conducted under a variety of conditions. Secondly, various olefins can be used as suitable substrates, including activated and inactive polysubstituted olefins. Third, almost all mercaptans can be used for reactions, including highly functional substances. Finally, this reaction is very rapid, and the reaction conditions are mild, which can be conducted in the air environment [25,26,27]. The “thiol–ene “click reaction can be initiated by free radicals, initiated by ultraviolet light and free radicals using natural light, red light and redox system. The click reaction is fast and can be performed under normal temperature and pressure. If it is initiated by ultraviolet light, the reaction yield can reach more than 90% in a few seconds. As a means of constructing new materials, the “thiol–ene” click reaction has many advantages: olefin compounds are very rich, with high selectivity, and can synthesize a large number of compounds with various structures [28,29,30,31]. Recently, Jawerth reported that the ethanol−soluble fraction of Lignoboost Kraft lignin was selectively allylated using allyl chloride by means of a mild and industrially scalable procedure. The obtained modified lignin was then subsequently cross−linked to prepare thermosetting resin via thermally induced thiol–ene chemistry [29]. Cao et al. prepared thermosetting lignin−based polyurethane coatings with superior corrosion resistance and a high content of lignin by the polymerization of lignin−based polyol. Firstly, the phenolic hydroxyls of enzymatic hydrolysis lignin were, firstly, selectively converted to primary aliphatic hydroxyls by an allylation reaction. Subsequently, the thermal radical initiated thiol–ene click reaction was applied to efficiently prepare the lignin−based polyol [30]. Zeng et al. developed durable a superhydrophobic and oleophobic coating based on perfluorodecanethiol fluorosilicone polyurethane (PFDT−FSPU) and thiol−modified cellulose substrate. The cross−linked network structure was formed by the radical polymerization of double bonds in PFDT−FSPU when the polyurethane was irradiated with ultraviolet light, and it was anchored on the surface of the cotton fibers by click reaction between the thiol−modified cellulose substrate and PFDT−FSPU. The coated fabric showed excellent durability and can still maintain superhydrophobicity and oleophobicity even after 600 cycles of abrasion or 30 times of washing cycles or 168 h of accelerated aging test [31].

The chemical modification of ethyl cellulose and lignin to prepare olefin−functionalized lignin and ethyl cellulose can effectively increase reaction activity and enrich their application performance. The chemical modification of ethyl cellulose and lignin to construct new functional materials has become a major strategy to increase their added value. In this study, ethyl cellulose (EC) and lignin are modified via esterification to prepare olefin−functionalized ethyl cellulose and lignin, which are further used to prepare cellulose and lignin cross−linker polymers via thiol–ene click chemistry. The chemical structure of olefin−functionalized EC and lignin, as well as ethyl cellulose and lignin cross−linked polymers are characterized. In addition, the thermal stability, microstructure, mechanical property and pyrolysis gas composition are also investigated.

## 2. Experimental Procedure

### 2.1. Materials

Lignin (Hydroxyl content: 5.6 mmol/g), EC (Mw = 151,237, Mw unit = 454.5 g/mol, DP = 332, Hydroxyl content: 1.57–1.82 mmole/g), 1−Hydroxycyclohexyl phenyl ketone, undecylenic acid, 4−Dimethylaminopyridine (DMAP), trimethylacetic anhydride, dichloromethane (DCM), methanol, tetrahydrofuran (THF), ketone, azodiisobutyronitrile (AIBN), 3,6−Dioxa−1,8−octanedithiol and pentaerythritol tetra(3−mercaptopropionate) were obtained from commercial resources and used as received, unless otherwise mentioned.

### 2.2. Preparation of Olefin−Functionalized Ethyl Cellulose (OFE)

**Scheme 1:**
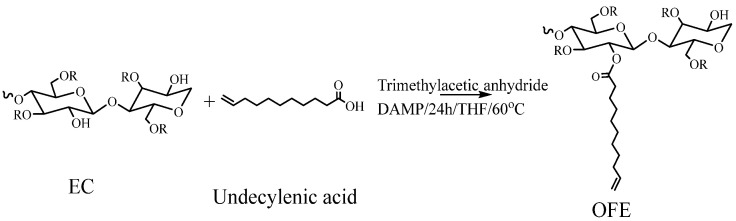
Synthesis of OFE.

EC (3.0 g, 0.01975 mol), undecylenic acid (1.82 g, 0.009878 mol), DMAP (12 mg, 0.09878 mmol) and trimethylacetic anhydride (1.83 g, 0.009878 mol) were dissolved in 50 mL dry THF and stirred at 60 °C under a nitrogen atmosphere for 48 h. In order to remove the unreacted undecylenic acid, the purification process was conducted by repeating dissolution into dry tetrahydrofuran and precipitation from methanol. The solid product was dried under vacuum to obtain OFE and label as EC−1−0.5. When the mole ratios of EC, undecylenic acid, DMAP and trimethylacetic anhydride were 1:1:0.001:1 and 1:1.3:0.001:1, the obtained OFEs were labeled as EC−1−1 and EC−1−1.3, respectively. Scheme 1 shows the Synthesis of OFE.

### 2.3. Preparation of Olefin−Functionalized Lignin (OFL)

**Scheme 2:**
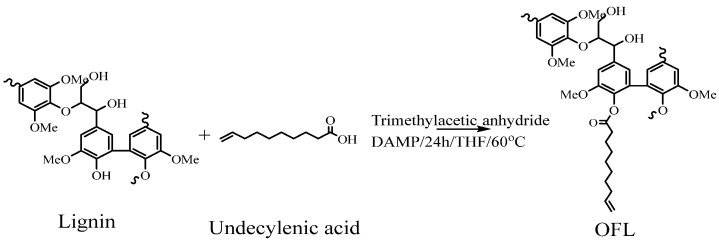
Synthesis of OFL.

Lignin (5.0 g, 28.75 mmol), undecylenic acid (2.645 g, 14.375 mmol), DMAP (17.53 mg, 0.14375 mmol) and trimethylacetic anhydride (2.677 g, 14.375 mmol) were dissolved in 50 mL dry THF. The mixture was stirred at 60 °C under a nitrogen atmosphere for 48 h. In order to remove the unreacted undecylenic acid, the purification process was conducted by repeating dissolution into dry tetrahydrofuran and precipitation from methanol. The solid product was dried under vacuum to obtain OFLs and labeled lignin−1−0.5. When the mole ratios of lignin, undecylenic acid, DMAP and trimethylacetic anhydride were 1:1:0.001:1 and 1:1.3:0.001:1, the obtained OFLs were labeled as lignin−1−1 and lignin−1−1.3, respectively. Scheme 2 shows the synthesis of OFL.

### 2.4. Determination of Olefin Group Concentration in OFE and OFL

The olefin group concentration in OFE and OFL was investigated with the internal standard method using 1,2,4,5−tetrachloro−3−nitrobenzene as the interior label. OFE, OFL and 1,2,4,5−tetrachloro−3−nitrobenzene were dissolved in CDCl_3_, and the ^1^H NMR of the mixture was tested. The signals at 5.0 ppm and 5.82 ppm from the protons of the olefin group were also integrated. The signal at 7.75 ppm from the protons of 1,2,4,5−tetrachloro−3−nitrobenzene was 100. A detailed calculation is presented in [32].

### 2.5. Preparation of Wood−Derived Polymers through the Chemical Cross−Linking of OFE and OFL via Thiol–Ene Click Chemistry

**Scheme 3:**
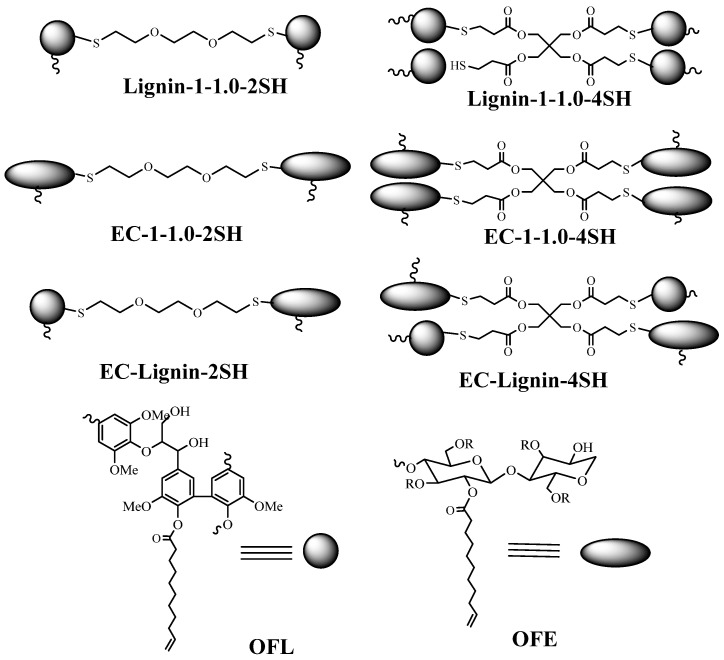
Schematic diagram of the chemical structure of the wood−derived polymers through the chemical cross−linking of OFE and OFL via thiol–ene click chemistry.

OFL or OFL polymers, thiol cross−linkers and 1−Hydroxycyclohexyl phenyl ketone (5% of total mass) were dissolved in dry THF. The mixture was stirred for 30 min and ultrasound for 20 min at room temperature. Then, the mixture was poured into a polytetrafluoroethylene mold, dried under tin foil for 24 h and vacuumed for 24 h at room temperature; then, a UV light was used for 10 min (two 10 W UV lamp tubes). The reactants and obtained wood−derived polymers are shown in Table 1. A schematic diagram of the chemical structure of the wood−derived polymers is presented in Scheme 3.

### 2.6. Characterization

Fourier transform infrared spectrometry (FTIR) spectra were obtained using a PerkinElmer spectrum 100 FTIR spectrometer. ^1^H NMR spectra were recorded on a Bruker Avance III HD 300 spectrometer using CDCl3 as the solvent with tetramethylsilane (TMS) as the internal reference. The gel content (C_gel_) was determined by Soxhlet extraction. First, approximately 0.5 g of polymer (recorded as m_0_) was precisely weighed, extracted with acetone for 24 h, dried in a vacuum oven at 50 °C for 24 h and weighed again (recorded as m_1_). The C_gel_ was calculated using (m_1_/m_0_) × 100%, and the three samples were tested for average. TG−FTIR spectra were analyzed by using a 409PC thermal analyzer coupled with a Nicolet IS10 FTIR instrument. The temperature was increased from 40 to 800 °C at a rate of 10 °C min^−1^ under a N_2_ atmosphere. Tensile tests were conducted on an E43.104 Universal Testing Machine (MTS Instrument Crop., Shenzhen, China). Dog−bone shaped polymers with a length of 20 mm and a width of 5.0 mm were tested at room temperature with a crosshead speed of 20 mm min^−1^. Five replicate samples were used to obtain an average value for each. The microstructure of the cross−linked polymer was determined using German Leica microscope DM750M.

## 3. Results and Discussion

**Figure 1:**
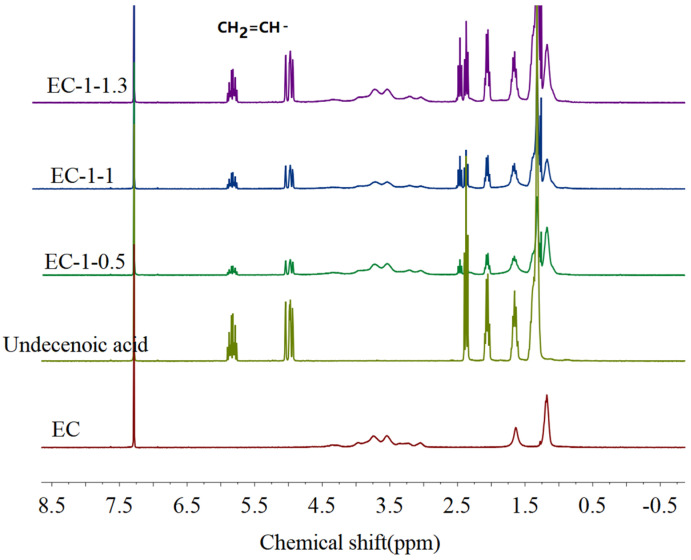
^1^H NMR of OFE, EC and undecenoic acid.

**Figure 2:**
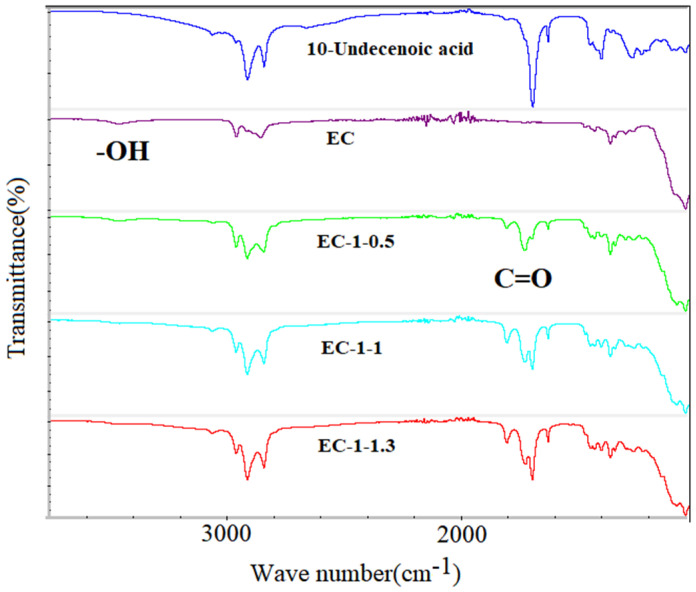
FT−IR of OFE, EC and undecenoic acid.

The esterification products of lignin and EC with undecenoic acid were characterized with FT−IR and ^1^H NMR, As seen in Figure 1, there are two strong proton signals at 5.0 ppm and 5.82 ppm in the ^1^H NMR of undecenoic acid, which are attributed to the protons of olefin [33,34]. There are no proton signals at 5.0 ppm and 5.82 ppm in the ^1^H NMR of EC. After esterification, proton signals at 5.0 ppm and 5.82 ppm gradually increased in the ^1^H NMR of EC−1−0.5, EC−1−1.0 and EC−1−1.3. The results indicate that OFE products were obtained. As seen in Figure 2, the strong peak at 1725 cm^−1^ is attributed to the infrared absorption peak of the carbonyl group in the FT−IR of undecenoic acid [35,36,37]. There is no peak at 1725 cm^−1^ in FT−IR of EC. After esterification, infrared absorption peaks of the carbonyl group gradually increased in the FT−IR of EC−1−0.5, EC−1−1.0 and EC−1−1.3, which indicates that the carbonyl groups were formed after esterification. The peak at 1675 cm^−1^ in the FT−IR of EC−1−0.5, EC−1−1.0 and EC−1−1.3 is attributed to the olefin groups [35,36,37]. The formation of carbonyl groups and the appearance of olefin groups suggests that OFE products were obtained.

**Figure 3:**
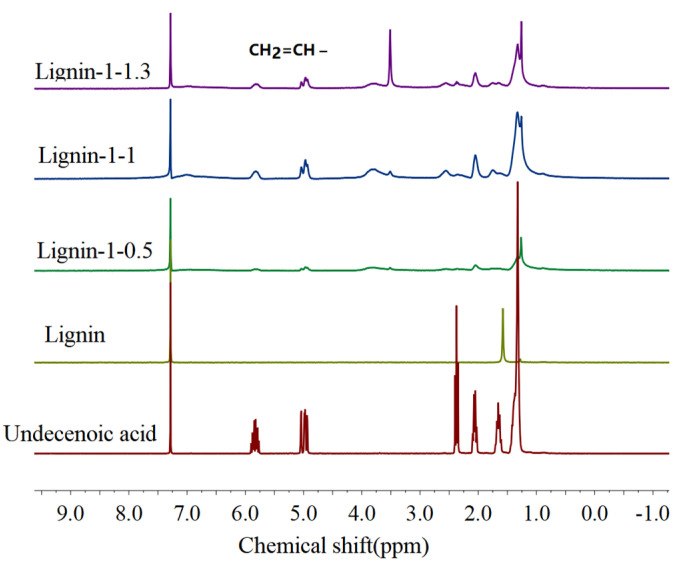
^1^H NMR of OFL, lignin and undecenoic acid.

**Figure 4:**
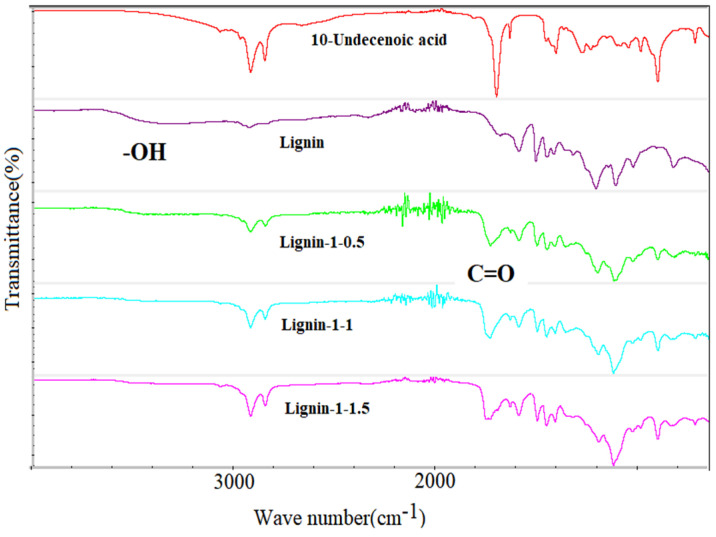
FT−IR of OFL, lignin and undecenoic acid.

The ^1^H NMR of OFL showed a similar characteristic absorption peak compared with that of OFE. As seen in Figure 3, there are no proton signals at 5.0 ppm and 5.82 ppm in the ^1^H NMR of lignin. After esterification, proton signals gradually increased at 5.0 ppm and 5.82 ppm in the ^1^H NMR of lignin−1−0.5, lignin−1−1.0 and lignin−1−1.3. The results indicate that OFL products were obtained. Similarly, the FT−IR of OFL showed a similar characteristic absorption peak compared that of with OFE. As seen from Figure 4, the strong peak at 1725 cm^−1^ is attributed to the infrared absorption peak of the carbonyl group in the FT−IR of undecenoic acid [33,34]. There is no peak at 1725 cm^−1^ in the FT−IR of lignin. After esterification, infrared absorption peaks of the carbonyl group gradually increased in the FT−IR of lignin−1−0.5, lignin−1−1.0 and lignin−1−1.3, which indicates that the carbonyl groups were formed after esterification. The peak at 1675 cm^−1^ in the FT−IR of lignin−1−0.5, lignin−1−1.0 and lignin−1−1.3 is attributed to olefin groups [35,36,37]. The formation of carbonyl groups and the appearance of olefin groups suggests that OFL products were obtained.

**Figure 5:**
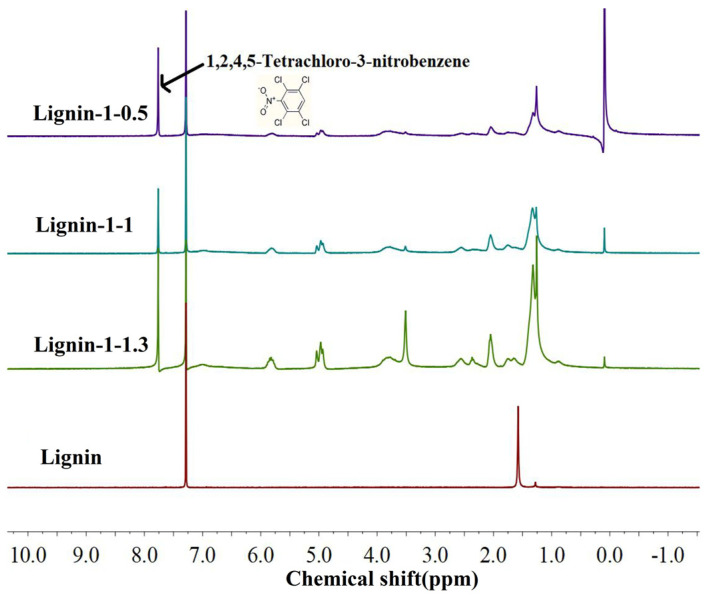
^1^H NMR of OFL with the interior label compared with lignin.

**Figure 6:**
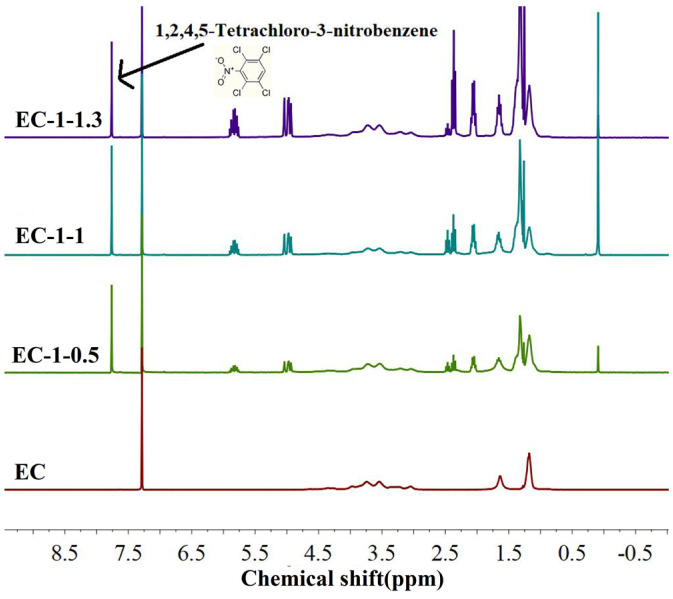
^1^H NMR of OFE with the interior label compared with EC.

The olefin group concentration in OFL and OFE was determined with the internal standard method using 2,3,5,6−Tetrachloro−3−nitrobenzene as the interior label. The ^1^H NMR of OFL and OFE with the interior label was detected and the results are shown in Figure 5 and Figure 6. The strong signal at 7.75 ppm in Figure 5 and Figure 6 is attributed to the protons of 2,3,5,6−Tetrachloro−3−nitrobenzene [38,39]. The olefin group concentration in OFL and OFE was calculated according to the procedure presented in a recent study [32]. The results are shown in Table 2, revealing that the olefin group concentration in OFL increased from 1.8060 to 2.8096 mmol/g when the n(−COOH):n(−OH) increased from 0.5 to 1.3. The olefin group concentration in OFE increased from 2.9200 to 3.7000 mmol/g when the n(−COOH):n(−OH) increased from 0.5 to 1.3.

**Figure 7:**
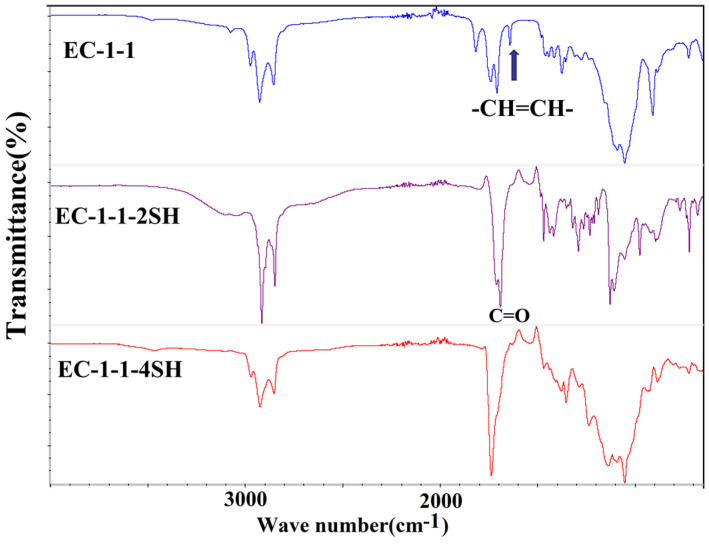
FT−IR of EC cross−linked polymers compared with that of EC−1−1.

**Figure 8:**
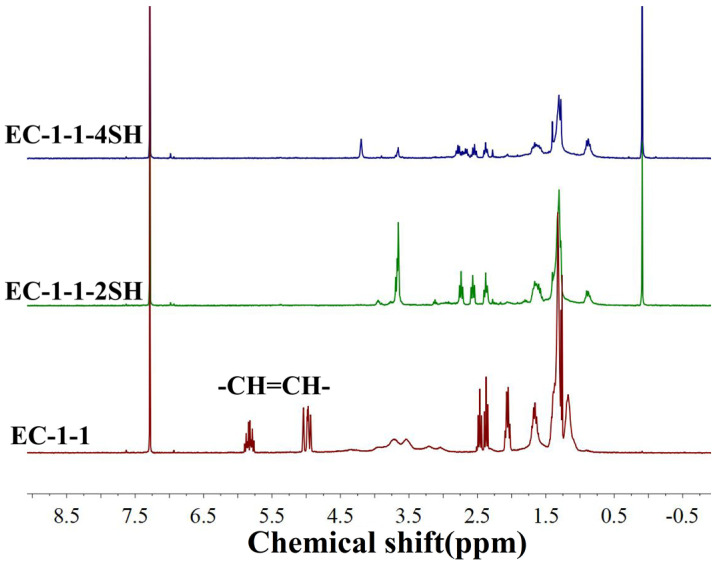
^1^H NMR of EC cross−linked polymer compared with that of EC−1−1.

The chemical structure of EC cross−linked polymers and lignin cross−linked polymers were investigated with FT−IR and ^1^H NMR. As seen from Figure 7, the FT−IR of EC−1−1−2SH and EC−1−1−4SH was compared with EC−1−1. The peak at 1675 cm^−1^ in the FT−IR of EC−1−1 is attributed to the olefin groups [35,36,37]. After the thiol–ene click reaction, there is no peak at 1675 cm^−1^ in the FT−IR of EC−1−1−2SH and EC−1−1−24H. The chemical structure of the dissolved part for EC cross−linked polymers was investigated with ^1^H NMR. As seen from Figure 8, the ^1^H NMR of EC cross−linked polymer was compared with that of EC−1−1. The two strong proton signals at 5.0 ppm and 5.82 ppm in the ^1^H NMR of EC−1−1 are attributed to the protons of olefin [33,34]. When the thiol–ene click reaction finished, there were no protons at 5.0 ppm and 5.82 ppm in the ^1^H NMR of EC−1−1−2SH and EC−1−1−4SH, which indicates that there was a thiol–ene click reaction between OFL and the cross−linker (3,6−Dioxa−1,8−octanedithiol, pentaerythritol tetra(3−mercaptopropionate)), and that the EC cross−linked polymers were obtained.

**Figure 9:**
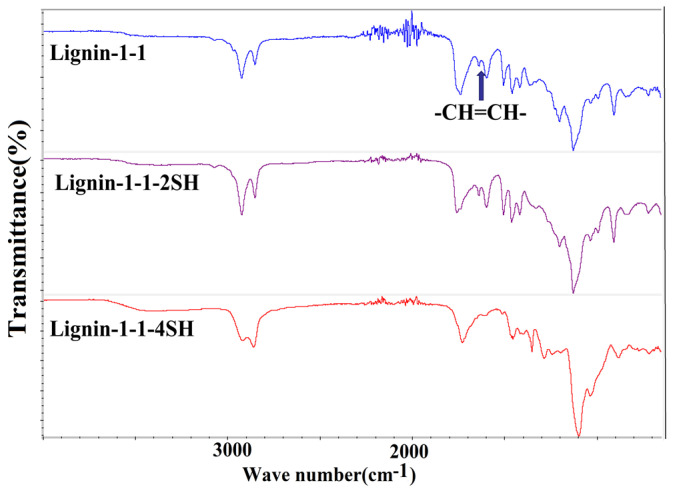
FT−IR of lignin cross−linked polymer compared with that of lignin.

**Figure 10:**
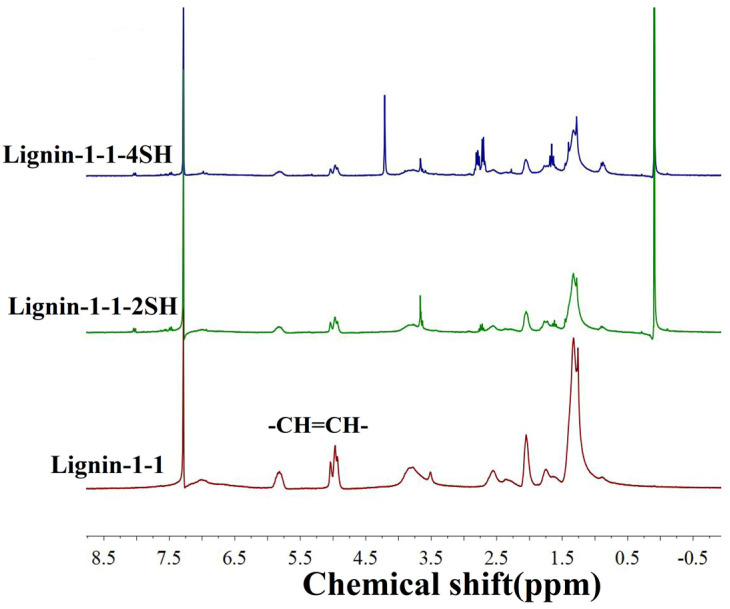
^1^H NMR of lignin cross−linked polymer compared with that of lignin.

Figure 9 shows the FT−IR of ligin−1−1−2SH and lignin−1−1−4SH compared with that of lignin−1−1. The peak at 1675 cm^−1^ in the FT−IR of ligin−1−1 is attributed to olefin groups [35,36,37]. When the thiol–ene click reaction finished, the peak at 1675 cm^−1^ in the FT−IR of ligin−1−1−2SH and ligin−1−1−4SH decreased obviously, which shows that there is still a small amount of the olefin group in the lignin cross−linked polymer. Figure 10 shows the ^1^H NMR of the chemical structure of the dissolved part for the lignin cross−linked polymers. The ^1^H NMR of the lignin cross−linked polymer was compared with that of lignin−1−1. The two strong proton signals at 5.0 ppm and 5.82 ppm in the ^1^H NMR of ligin−1−1 are attributed to the protons of olefin [34,34]. After the thiol–ene click reaction, the proton signals at 5.0 ppm and 5.82 ppm in the ^1^H NMR of lignin−1−1−2SH and lignin−1−1−4SH appeared weak and almost disappeared, which indicates that the thiol–ene click reaction between OFL and cross−linker (3,6−Dioxa−1,8−octanedithiol, pentaerythritol tetra(3−mercaptopropionate)) is incomplete, because the precise proportion of olefin and sulfhydryl is difficult to control.

**Figure 11:**
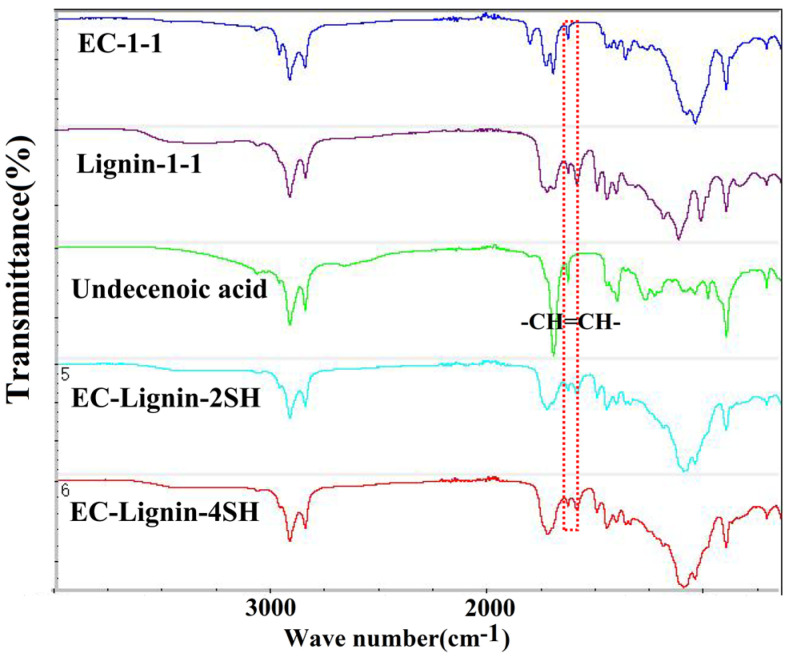
FT−IR of the EC–lignin cross−linked polymers compared with that of lignin−1−1, EC−1−1 and undecenoic acid.

**Figure 12:**
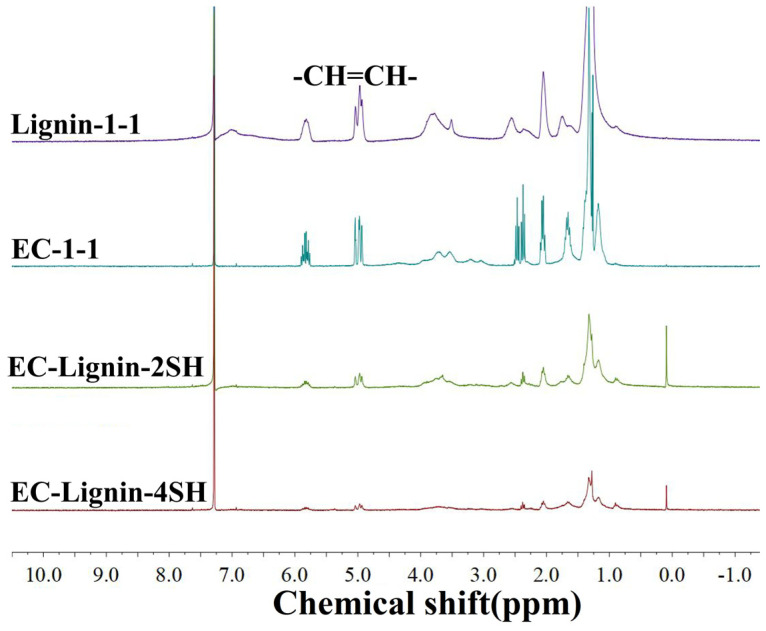
^1^H NMR of EC–lignin cross−linked polymer compared with those of lignin−1−1 and EC−1−1.

Figure 11 shows the FT−IR of the EC–lignin cross−linked polymer compared with that of lignin−1−1, EC−1−1 and undecenoic acid. When the thiol–ene click reaction occurred, the peak at 1675 cm^−1^ in the FT−IR of EC−ligin−2SH and EC−ligin−4SH was weaker than those of lignin−1−1 and EC−1−1, which shows that there is still a small amount of the olefin group in the EC–lignin cross−linked polymer. Figure 12 shows the ^1^H NMR of the chemical structure of the dissolved part for the EC–ligin cross−linked polymers, and the ^1^H NMR of the lignin cross−linked polymers compared with that of lignin−1−1 and EC−1−1. The proton signals at 5.0 ppm and 5.82 ppm in the ^1^H NMR of EC−ligin−2SH and EC−ligin−4SH were low, which was caused by the thiol–ene click reaction.

**Figure 13:**
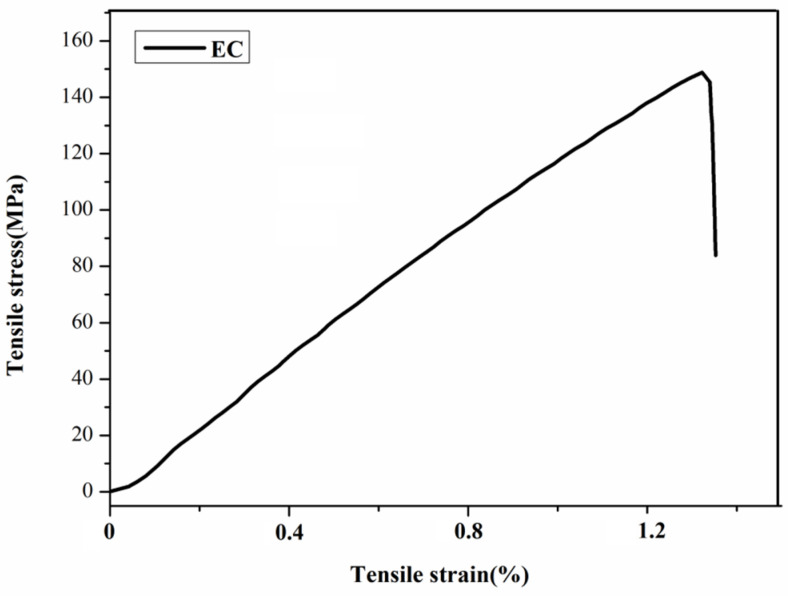
Tensile stress and strain of EC.

**Figure 14:**
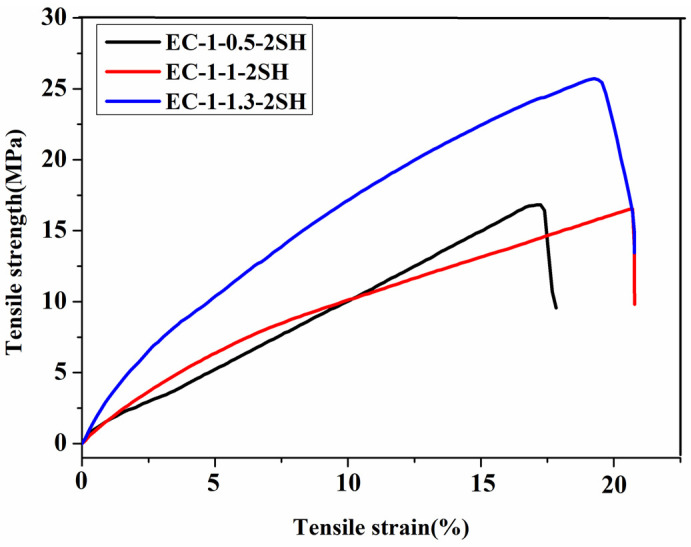
Tensile stress and strain of the EC cross−linked polymers using 3,6−Dioxa−1,8−octanedithiol as the cross−linker.

**Figure 15:**
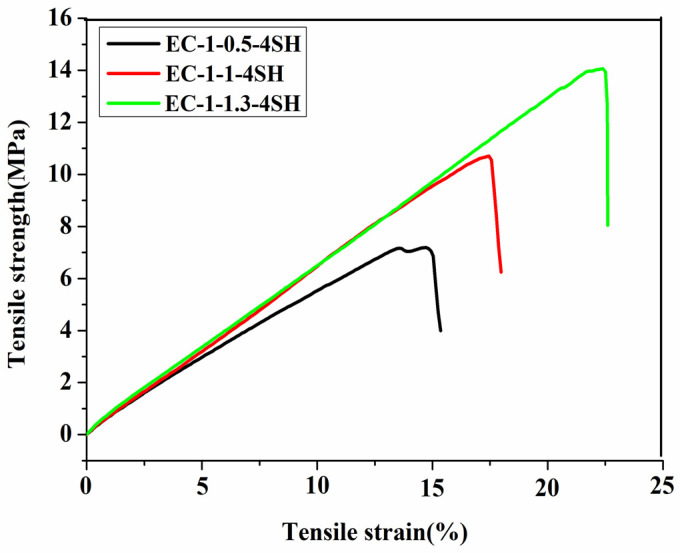
Tensile stress and strain of the EC cross−linked polymers using pentaerythritol tetra(3−mercaptopropionate) as the cross−linker.

**Figure 16:**
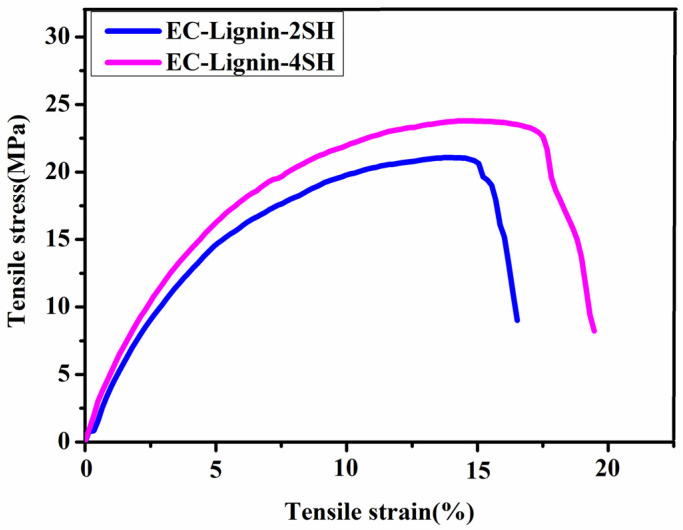
Tensile stress and strain of the EC–lignin cross−linked polymers.

The tensile stress and strain at break of the EC and cross−linked polymers were investigated and the results are shown in Figure 13, Figure 14, Figure 15 and Figure 16. As seen in Figure 13, the tensile stress and strain at break of EC were 146.8 MPa and 1.32%, respectively. For the cross−linked polymers, the tensile stress and strain at break increased when the olefin group concentration increased in OFE. When the olefin group concentration increased from 2.9200 to 3.7000 mmol/g, the tensile stress at break increased from 16.41 MPa to 23.59 MPa, as seen in Table 3, while the tensile strain at break firstly increased from 17.25% to 19.41% and then decreased to 18.12%. When the pentaerythritol tetra(3−mercaptopropionate) was used as cross−linker, as seen in Figure 15, the tensile stress at break increased from 6.86 MPa to 13.94 MPa, and the tensile strain increased from 14.85% to 22.38. EC−lignin−4SH showed excellent tensile properties compared to EC−lignin−2SH. The tensile stress at break for EC−lignin−4SH was 15.19 MPa, which is higher than that of EC−lignin−2SH at 13.72 MPa, and the tensile strain at break increased from 15.12% to 17.25%. The gradual enhancement in mechanical properties is positively correlated with the olefin group concentration. The mechanical properties of the lignin cross−linked polymers (lignin−1−1−2SH and lignin−1−1−4SH) were not tested because the polymers were in powder form. Compared with EC, the tensile strength of the cross−linked polymers decreased significantly, but the tensile strain increased sharply, which is caused that the flexible aliphatic hydrocarbon chain from undecylenic acid as the branched chains of EC and lignin increased the distance among the main chains, weakened the interaction force among the main chains and reduced the hydrogen bond interaction in the matrix of EC and lignin. The flexible aliphatic hydrocarbon chain from undecylenic acid contributed to plasticization by functioning as an internal plasticizer.

The *C*_gel_ of the cross−linked polymers were detected and the results are shown in Table 3. The results show that *C*_gel_ is positively correlated to the tensile stress for the same type of polymers. For the EC−n−2SH cross−linked polymers, when the tensile stress increased from 16.41 MPa to 23.59 MPa, the *C*_gel_ increased from 94.9% to 98.0%. The other cross−linked polymers showed the same relationship between the gel content and the tensile strength.

**Figure 17:**
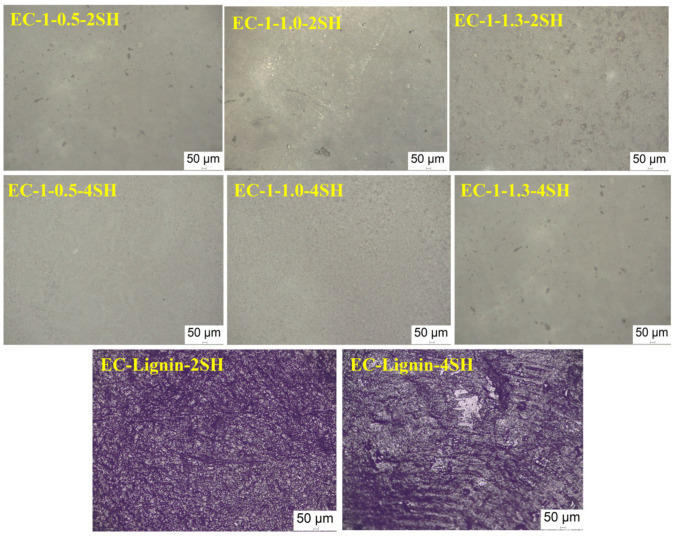
Microstructure of the cross−linked polymers.

The microstructure of the cross−linked polymers were investigated using a Leica DM750M optical microscope. As seen in Figure 17, the EC cross−linked polymers showed a uniform surface structure without large cracks or micropores, while the EC−lignin polymers showed an uneven surface structure with many large cracks and micropores, which may be due to solvent evaporation.

**Figure 18:**
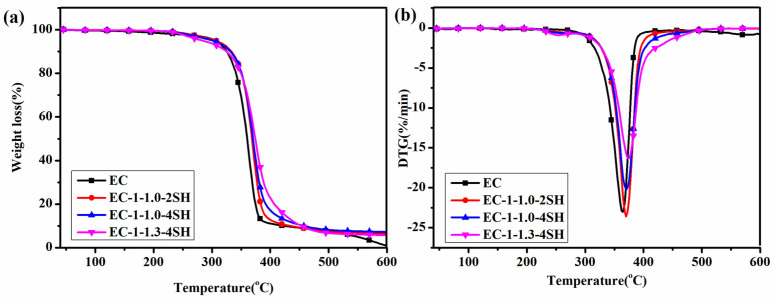
TGA (**a**) and DTG (**b**) curves of the cross−linked polymers.

Figure 18 shows the thermal stability of the EC and cross−linked polymers. Only one thermal degradation stage occurred at 360–370 °C for EC and all cross−linked polymers. It has been reported that, when cellulose−based polymers are degraded at high temperatures, their polymerization degree is reduced, the chemical composition also changes and the carbonyl groups increase [40,41]. When cellulose−based polymers are fully degraded, carbon monoxide, carbon dioxide, ethylene, water and carbon are produced [42,43]. Table 4 shows the thermal degradation temperature (T_d_), the peak value of the thermal degradation temperature (T_P_) and the char residue of the cross−linked polymers. The thermal stability of the cross−linked polymers (EC−1−1.0−2SH, EC−1−1.0−4SH and EC−1−1.3−4SH) was higher than that of EC. The T_d_, T_P_ and char residue of the cross−linked polymers increased compared with those of EC. This is because OFE and the cross−linkers, such as 3,6−Dioxa−1,8−octanedithiol and pentaerythritol tetra(3−mercaptopropionate), contain thermostable ester groups, which makes the cross−linked polymers difficult degrade.

**Figure 19:**
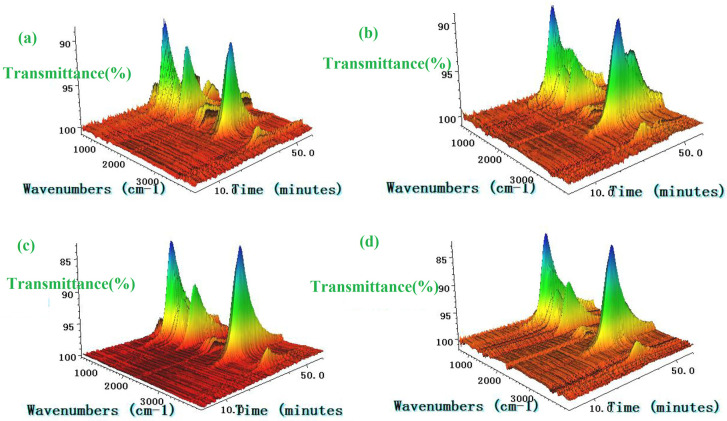
Three−dimensional FT−IR of EC (**a**), EC−1−1.0−2SH (**b**), EC−1−1.0 − 4SH (**c**) and EC−1−1.3−4SH (**d**).

**Figure 20:**
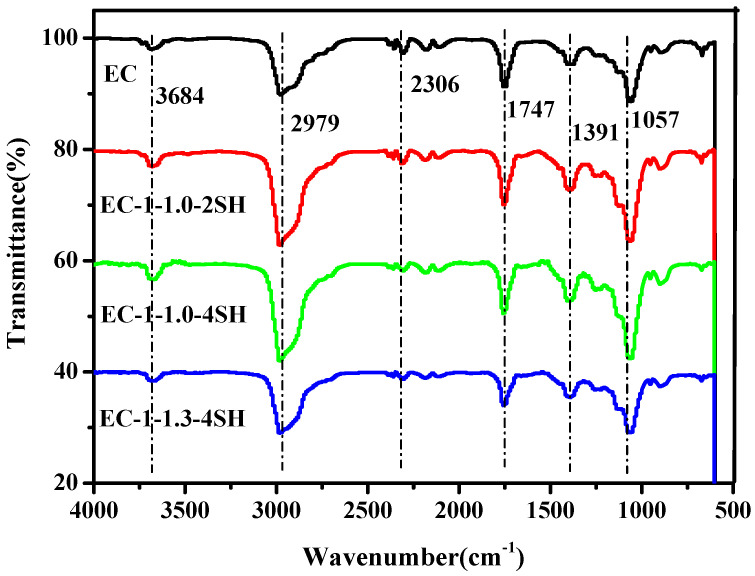
Two−dimensional FT−IR of EC, EC−1−1.0−2SH, EC−1−1.0−4SH and EC−1−1.3−4SH.

In order to further investigate the composition of the thermal degradation products, TGA−FTIR was carried out. Figure 19 and Figure 20 show the 3D and 2D FT−IR, respectively, of EC (a), EC−1−1.0−2SH (b), EC−1−1.0−4SH (c) and EC−1−1.3−4SH (d). The infrared characteristic absorption peak of the gas phase of the thermal degradation products can be clearly observed in Figure 20. The data were collected at the fastest decomposition temperature of 340 °C. The infrared characteristic absorption peaks at 3684, 2979, 2306 and 1747, 1391, 1057 cm^−1^ were attributed to H_2_O, aliphatic hydrocarbon segments, CO_2_ and degradation products containing ester groups, respectively [44,45,46]. The existence of ester groups in the cross−linked polymers and degradation products make them more thermally stable.

## 4. Conclusions

In this study, ethyl cellulose and lignin were modified via esterification to prepare olefin−functionalized ethyl cellulose and lignin, which were further used to prepare cellulose and lignin cross−link polymers via thiol–ene click chemistry. The results show that the olefin group concentration in the olefin−functionalized ethyl cellulose and lignin reached 2.8096 mmol/g and 3.7000 mmol/g, respectively. The tensile stress at break of the cellulose cross−linked polymers reached 23.59 MPa. The gradual enhancement in the mechanical properties was positively correlated with the olefin group concentration. Compared with EC, the tensile strength of the cross−linked polymers decreased significantly, but the tensile strain increased sharply. The flexible aliphatic hydrocarbon chain from undecylenic acid contributed to plasticization by functioning as an internal plasticizer. The changes in mechanical properties make the cross−linked polymers easier to process, expanding their application range. The EC–lignin polymers had an uneven surface structure and there were many large cracks and micropores due to the rigid benzene ring structure of lignin. The infrared characteristic absorption peaks at 3684, 2979, 2306 and 1747, 1391, 1057 cm^−1^ indicated that H_2_O, aliphatic hydrocarbon segments, CO_2_ and degradation products containing ester groups were released. The existence of ester groups in the cross−linked polymers and degradation products makes them more thermally stable. The obtained ethyl cellulose and lignin cross−link polymers are of great significance for practical applications and contribute towards the high−value−added utilization of lignin and cellulose.

## Figures and Tables

**Table 1:** Reactants and the obtained wood−derived polymers.

Cross−linked Polymer	Reactants
EC−1−0.5−2SH	EC−1−0.5 and 3,6−Dioxa−1,8−octanedithiol, n(−CH=CH−) = n(−SH)
EC−1−1.0−2SH	EC−1−1.0 and 3,6−Dioxa−1,8−octanedithiol, n(−CH=CH−) = n(−SH)
EC−1−1.3−2SH	EC−1−1.3 and 3,6−Dioxa−1,8−octanedithiol, n(−CH=CH−) = n(−SH)
EC−1−0.5−4SH	EC−1−0.5 and pentaerythritol tetra(3−mercaptopropionate), n(−CH=CH−) = n(−SH)
EC−1−1.0−4SH	EC−1−1.0 and pentaerythritol tetra(3−mercaptopropionate), n(−CH=CH−) = n(−SH)
EC−1−1.3−4SH	EC−1−1.3 and pentaerythritol tetra(3−mercaptopropionate), n(−CH=CH−) = n(−SH)
EC−Lignin−2SH	EC−1−1, Lignin−1−1, and 3,6−Dioxa−1,8−octanedithiol, n(−CH=CH−)/n(−CH=CH−)/n(−SH) = 0.5:0.5:1
EC−Lignin−4SH	EC−1−1, lignin−1−1, and pentaerythritol tetra(3−mercaptopropionate), n(−CH=CH−)/n(−CH=CH−)/n(−SH) = 0.5:0.5:1
Lignin−1−1−2SH	Lignin−1−1 and 3,6−Dioxa−1,8−octanedithiol, n(−CH=CH−) = n(−SH)
Lignin−1−1−4SH	Lignin−1−1 and pentaerythritol tetra(3−mercaptopropionate), n(−CH=CH−) = n(−SH)

**Table 2:** Olefin group concentration in OFL and OFE.

OFL and OFE Samples	Olefin Group Concentration in OFL and OFE (mmol/g)
Lignin−1−0.5	1.8060
Lignin−1−1	2.3818
Lignin−1−1.3	2.8096
EC−1−0.5	2.9200
EC−1−1	3.5873
EC−1−1.3	3.7000

**Table 3:** Tensile stress, tensile strain and *C*_gel_ of the cross−linked polymers.

Polymer Films	Tensile Stress (MPa)	Tensile Strain (%)	*C*_gel_ (%)
EC	146.89 ± 10.24	1.32 ± 0.02	−
EC−1−0.5−2SH	16.41 ± 1.22	17.25 ± 0.19	94.9 ± 0.8
EC−1−1.0−2SH	16.53 ± 1.10	19.41 ± 2.18	96.4 ± 0.4
EC−1−1.3−2SH	23.59 ± 3.01	18.12 ± 0.13	98.0 ± 0.4
EC−1−0.5−4SH	6.86 ± 0.21	14.85 ± 1.11	92.7 ± 0.6
EC−1−1.0−4SH	10.55 ± 0.89	16.92 ± 1.76	94.3 ± 0.7
EC−1−1.3−4SH	13.94 ± 1.00	22.38 ± 3.20	95.2 ± 0.9
EC−Lignin−2SH	13.72 ± 0.02	15.12 ± 1.19	94.8 ± 0.2
EC−Lignin−4SH	15.19 ± 1.03	17.25 ± 2.10	97.2 ± 0.5
Lignin−1−1−2SH	−	−	−
Lignin−1−1−4SH	−	−	−

**Table 4:** T_d,_ T_P_ and char residue of the cross−linked polymers.

Cross−linked Polymers	T_d_ (°C)	T_P_ (°C)	Char Residue (%)
EC	339.8	364.1	0.9
EC−1−1.0−2SH	348.2	370.2	6.6
EC−1−1.0−4SH	346.8	371.2	7.2
EC−1−1.3−4SH	344.0	374.1	5.7

## Data Availability

Not applicable.
